# Context-sensitive trace inlining for Java^☆^

**DOI:** 10.1016/j.cl.2013.04.002

**Published:** 2013-12

**Authors:** Christian Häubl, Christian Wimmer, Hanspeter Mössenböck

**Affiliations:** a Institute for System Software, Christian Doppler Laboratory for Automated Software Engineering, Johannes Kepler University Linz, Altenbergerstrasse 69, 4040 Linz, Austria; b Oracle Labs, 500 Oracle Parkway, Redwood Shores, CA 94065, USA

**Keywords:** Java, Just-in-time, Trace-based, Compilation, Inlining

## Abstract

Method inlining is one of the most important optimizations in method-based just-in-time (JIT) compilers. It widens the compilation scope and therefore allows optimizing multiple methods as a whole, which increases the performance. However, if method inlining is used too frequently, the compilation time increases and too much machine code is generated. This has negative effects on the performance.

Trace-based JIT compilers only compile frequently executed paths, so-called traces, instead of whole methods. This may result in faster compilation, less generated machine code, and better optimized machine code. In the previous work, we implemented a trace recording infrastructure and a trace-based compiler for ${\mathrm{Java}}^{\mathrm{TM}}$, by modifying the Java HotSpot VM. Based on this work, we evaluate the effect of trace inlining on the performance and the amount of generated machine code.

Trace inlining has several major advantages when compared to method inlining. First, trace inlining is more selective than method inlining, because only frequently executed paths are inlined. Second, the recorded traces may capture information about virtual calls, which simplify inlining. A third advantage is that trace information is context sensitive so that different method parts can be inlined depending on the specific call site. These advantages allow more aggressive inlining while the amount of generated machine code is still reasonable.

We evaluate several inlining heuristics on the benchmark suites DaCapo 9.12 Bach, SPECjbb2005, and SPECjvm2008 and show that our trace-based compiler achieves an up to 51% higher peak performance than the method-based Java HotSpot client compiler. Furthermore, we show that the large compilation scope of our trace-based compiler has a positive effect on other compiler optimizations such as constant folding or null check elimination.

## Highlights

• We describe trace inlining for Java and compare it to method inlining.
• We implemented trace inlining for the Java HotSpot VM.
• We present multiple trace inlining heuristics for our trace-based JIT compiler.
• We evaluate the impact of our trace inlining heuristics on several benchmarks.
• We evaluate how trace inlining affects other compiler optimizations.

## 1 Introduction

**Fig. 1:**
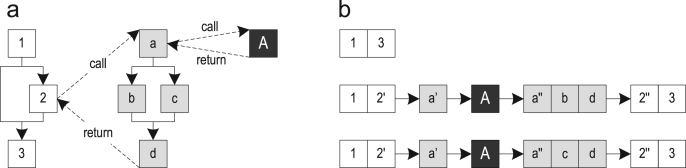
Possible traces through three methods: (a) control flow graphs and (b) possible traces.

Method-based just-in-time (JIT) compilation translates whole methods to optimized machine code, while trace-based compilation uses frequently executed paths, so-called traces, as the compilation unit [1]. This can increase the peak performance, while reducing the amount of generated machine code. Fig. 1 shows the control flow graphs (CFGs) of three methods as well as three possible traces through them. The start of a trace is called a *trace anchor*, which is block 1 for all traces in the example. It highly depends on the specific trace recording implementation which blocks are chosen as trace anchors.

In a virtual machine (VM), traces can be recorded by instrumenting bytecode execution. Those traces are then compiled to optimized machine code. If a method part that was not compiled has to be executed, it is common to fall back to the interpreter.

Most existing trace recording implementations allow traces to cross method boundaries [1], [2], [10], [12], [18]. This may result in large traces that must be compiled together.

In the previous work [14], [15], we implemented a trace-based JIT compiler based on Oracle's ${\mathrm{Java}}^{\mathrm{TM}}$ HotSpot client compiler [19]. Our earlier conference paper [15] focused on trace inlining and contributed the following:

• We described how to perform trace inlining and discuss its advantages compared to method inlining.
• We presented multiple trace inlining heuristics implemented for our trace-based JIT compiler.
• We evaluated the impact of our trace inlining heuristics on compilation time, peak performance, and amount of generated machine code for the DaCapo 9.12 Bach [3] benchmark suite.

This paper is an extended version of our earlier conference paper [15], and contributes the following new aspects:

• We present our trace recording and our trace inlining approaches in more detail.
• We describe how compiler intrinsics for native methods can profit from the larger compilation scope that is achieved by our trace inlining.
• We additionally evaluate our inlining heuristics on the benchmark suites SPECjbb2005 [23] and SPECjvm2008 [24]. Furthermore, we also compare the peak performance of our best trace inlining heuristic to the Java HotSpot server compiler.
• We evaluate which high-level compiler optimizations do benefit from trace inlining due to the widened compilation scope.

The remaining paper is organized as follows: Section 2 gives a short overview of our trace-based Java HotSpot VM. In Section 3 we illustrate our trace recording system, and in Section 4 we explain how we perform trace inlining. Section 5 presents different trace inlining heuristics. Section 6 discusses the benchmark results. In Section 7 we discuss related work, and Section 8 concludes the paper.

## 2 Overview

**Fig. 2:**
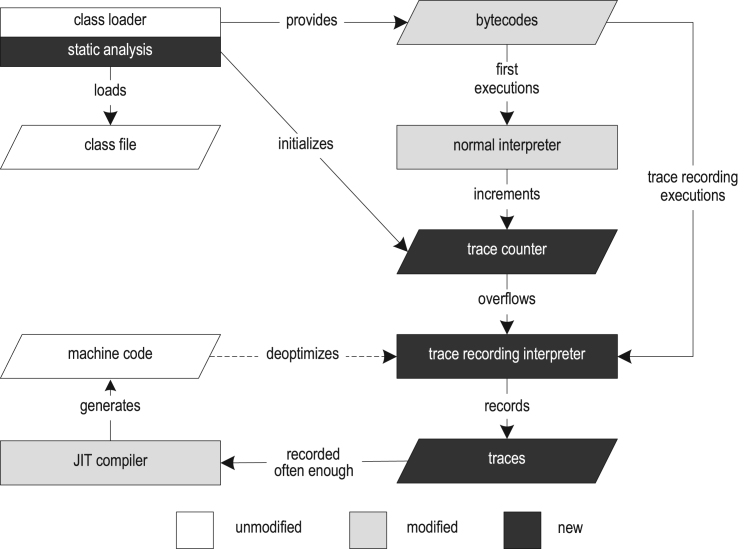
Structure of the tracing VM.

In the previous work, we implemented a trace recording infrastructure and a trace-based JIT compiler for Java [14], [15]. Fig. 2 shows the structure of our VM. Execution starts with the class loader that loads, parses, and verifies the class files. The class loader provides run-time data structures such as the constant pool and method objects to other parts of the VM. After class loading, a bytecode preprocessing step is performed that detects loops and creates tracing-specific data structures.

For trace recording, the Java HotSpot VM template interpreter [13] is duplicated and instrumented. This results in a normal and a trace recording interpreter. The normal interpreter executes bytecodes with nearly the same speed as the interpreter of the unmodified VM and is used for the initial executions. Whenever the normal interpreter encounters a trace anchor, it increments the invocation counter of that trace anchor. When the counter overflows, the trace anchor is marked as hot and execution switches to the trace recording interpreter. The current implementation supports two different kinds of traces: loop traces anchored at loop headers, and method traces anchored at method entries.

Oracle's Java HotSpot VM ships with two different JIT compilers that share most parts of the VM infrastructure. The *client compiler* is designed for startup performance and implements basic optimizations to achieve a decent peak performance [19]. Upon compilation, the compiler generates the high-level intermediate representation (HIR), which is in static single assignment (SSA) form [7] and represents the control flow graph. During and after building the HIR, optimizations such as constant folding, null check elimination, and method inlining are applied. The optimized HIR is translated to the low-level intermediate representation (LIR), which is close to machine code but still mainly platform independent. The LIR is then used for linear scan register allocation [27] and code generation.

The *server compiler* performs significantly more optimizations than the client compiler and produces highly efficient code to reach the best possible peak performance [21]. It is designed for long-running server applications where the initial JIT compilation constitutes only a small overhead in comparison to the total execution time. The server compiler uses the following compilation phases: parsing, machine-independent optimization, instruction selection, global code motion and scheduling, graph coloring register allocation, peephole optimization, and code generation. Some additional optimizations that the server compiler performs are loop-invariant code motion, loop unrolling, and escape analysis.

Our trace-based JIT compiler is based on the HotSpot client compiler. While our techniques are general enough to be applicable to the server compiler as well, the complex structure of the server compiler is less approachable for the changes that are required for trace-based compilation, especially in the context of a research project. Therefore, we decided to use the client compiler as our base.

When traces have been recorded often enough, our compiler at first merges the recorded traces into a trace graph. This data structure is a hybrid between a control flow graph and a trace tree [10], so that merge points may exist but paths may still be duplicated if advantageous. On this level, we perform general and tracing-specific optimizations such as constant folding, aggressive trace inlining, and explicit control flow duplication. The generated machine code is then directly invoked by the interpreters or by other compiled traces.

If a precondition for an aggressive optimization is violated during execution, our system deoptimizes [17] to the trace recording interpreter. Deoptimization at first saves all values that are live in the current compiled frame and then replaces that compiled frame with one or more interpreter frames. The exact number of created interpreter frames, depends on the inlining depth of the currently executed instruction. Then, the interpreter frames are filled with the previously saved values and execution continues in the trace recording interpreter.

When the trace recording interpreter takes over, it can record a partial trace that directly starts at the point of deoptimization instead of at the trace anchor. To detect too frequent deoptimization of compiled code, a counter is incremented every time a deoptimization occurs. After reaching a threshold, the compiled machine code is invalidated and another compilation is triggered that uses the originally recorded traces and all partial traces. This allows increasing method coverage or disabling specific aggressive optimizations, which in turn reduces the deoptimization frequency.

## 3 Trace recording

Our trace recording approach restricts traces to span at most one method [14]. When a trace anchor has been executed frequently enough, execution switches from the normal to the trace recording interpreter. For trace recording, every thread holds a tracing stack that contains the traces that are currently being recorded. Information about instructions that modify the control flow is stored in the topmost trace of the tracing stack, and the tracing stack is modified as necessary.

When a method invocation is reached during trace recording, the invocation is recorded in the caller trace. For virtual method invocations, we also record the receiver class. Upon entering the callee, a new method trace is pushed on the tracing stack and recording continues there.

When the callee returns, we pop the corresponding trace from the tracing stack and store it in a trace repository. Then, we link the caller and the callee trace by storing a pointer to the callee's trace in the caller's trace and continue recording for the caller. The linking preserves context-sensitive call information over method boundaries and results in a data structure that is similar to a dynamic call graph.

When a previously stored trace is recorded again, only a counter is incremented in the already stored trace instead of storing the trace another time. We consider traces to be different if they took different paths or if they invoked different callee traces. So, trace linking allows us to record exact call information for every executed path through the whole application. To reduce the number of recorded traces to a reasonable amount, we do not link loop traces and recursive method traces to their parent trace. After trace recording was performed a certain number of times for a trace anchor, we assume that all important traces for this anchor have been recorded and compile those traces to optimized machine code.

**Fig. 3:**
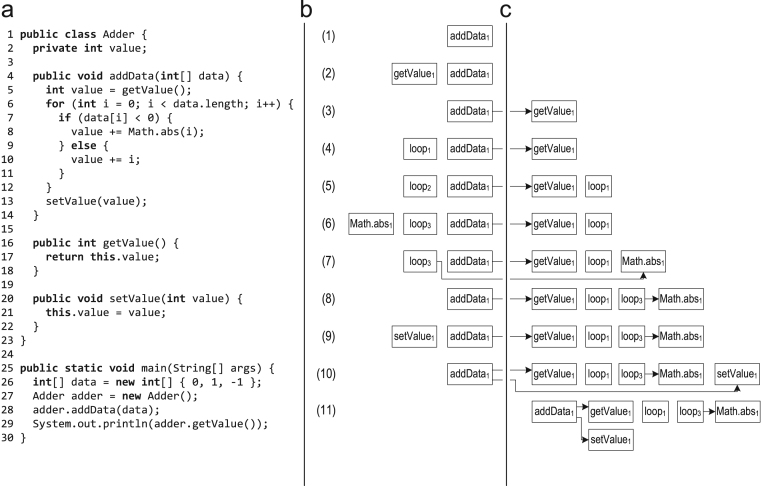
Tracing stack while trace recording: (a) source code; (b) tracing stack; and (c) traces recorded in the trace repository.

Fig. 3 shows a trace recording example where trace recording is triggered for the method addData(). (1) When the trace anchor at the method entry of addData() is marked as hot, execution switches to the trace recording interpreter and a method trace is pushed on the tracing stack. The method is executed from the beginning up to the invocation of the virtual method getValue(). When doing the virtual call, the invocation and the receiver class are stored in the caller trace. (2) Upon entering the method getValue(), a new method trace is pushed on the tracing stack and trace recording continues there. (3) When getValue() returns, the corresponding trace is popped from the tracing stack and stored in the trace repository. Then, the traces are linked by storing a pointer to the trace of getValue() in the trace of addData(). Execution and trace recording continues for addData() and reaches the loop header. (4) For recording the loop, a new loop trace is pushed on the tracing stack. (5) After the first loop iteration, when execution is back at the loop header, the loop trace is popped from the tracing stack and stored. For the next loop iteration, a new loop trace is pushed on the tracing stack. The second loop iteration executes the same path as the first iteration, so the system recognizes that the same trace was already recorded and does not store it again but only increments the counter within the previously recorded trace. (6) The third loop iteration takes a different path so that the method Math.abs() is invoked for which a new method trace is pushed on the tracing stack. (7) When Math.abs() returns, the corresponding trace is stored and linked to its caller trace. (8) Then, execution reaches the loop header and the loop exits. So, the loop trace is popped from the tracing stack and stored. (9) After the loop, the virtual method setValue() is invoked. So, the invocation and the receiver class are stored in the caller trace, and a new method trace is pushed on the tracing stack upon entering setValue(). (10) When setValue() returns, the corresponding trace is popped from the tracing stack, stored, and linked to its caller trace. (11) Eventually, the method addData() returns so that also this trace is popped from the tracing stack and stored. After that, the tracing stack is empty and execution switches back to the normal interpreter.

In the example above, it was assumed that no traces had been compiled for the invoked methods and the loop. If traces for the method getValue() had already been compiled earlier, the invocation of getValue() would execute the compiled machine code instead of interpreting the method. So, the trace recording interpreter can neither push a new method trace on the tracing stack, nor can it record any control flow in the invoked method. In that case, our trace recording approach does not preserve exact control flow information over method boundaries. It would be possible to not invoke compiled code and instead force this code to be executed in the trace recording interpreter if a trace is currently being recorded. However, this would drastically reduce the startup performance because the application would be interpreted for a significantly longer time.

For best-possible trace recording performance, all frequently executed operations (such as recording information for specific instructions) are directly implemented in the assembler templates of the trace recording interpreter. More complex operations, such as storing the recorded traces, are implemented in the C-based runtime of the interpreter. Our trace recording infrastructure also supports efficient multi-threading so that every Java thread can switch between the normal and the trace recording interpreters independently. Each thread uses a thread-local buffer for trace recording to achieve the best-possible trace recording performance.

During trace recording, multiple threads may operate on the data structure that holds the recorded traces. We observed that for most trace anchors, only a small number of traces is recorded so that storing a new trace is required rarely, while in most cases only the execution count of an already recorded trace is incremented. Therefore, we store the recorded traces in a data structure that avoids locks and atomic instructions when data is read. When it seems that a new trace was found, we lock our data structure for other writing threads and recheck under the lock if this trace is really new before adding it to the recorded traces. So, for the most frequent case, we can avoid synchronization and atomic machine instructions, which significantly increases the trace recording performance for multi-threaded applications.

## 4 Trace inlining

Method inlining replaces calls with copies of the actually called code. The inlining heuristics can be categorized into static and dynamic approaches.

The Java HotSpot client compiler uses a simple, static method inlining heuristic, where the method size is compared to a fixed limit. Virtual methods are inlined using static class hierarchy analysis (CHA) [8]. This analysis determines if a method is not overridden by any loaded subclass, in which case it can be inlined optimistically. If a subclass is loaded later on that overrides an optimistically inlined method, the generated machine code is invalidated. Dynamic inlining heuristics use profiling information to decide if a call is worth inlining.

Our trace-based JIT compiler supports both static and dynamic inlining heuristics by making use of the recorded trace information. Similar to method inlining, trace inlining also replaces calls with copies of the actually called code. This increases the compilation scope and may result in a higher performance.

### 4.1 Advantages of trace inlining

Trace inlining has several advantages over method inlining:

• Trace inlining does only inline frequently executed traces instead of whole methods. Method-based compilers try to use profiling information to avoid compilation of infrequently executed method parts [9], [25], [26]. This achieves a similar effect to trace inlining but is a complementary approach.
• The recorded traces contain context-sensitive information about which method parts are used by which caller. This information is preserved over method boundaries and can be used to avoid inlining of method parts that were executed frequently in total but are not required for the current caller.
• Traces also store information about the receivers of virtual calls and due to our trace linking, this information is also context sensitive. So, it might turn out that a certain call site invokes only methods of a specific receiver type. This information can be used for aggressive inlining of virtual methods. Method-based compilers also use profiling information for aggressive inlining of virtual calls, but in most compilers this information is not context sensitive.

### 4.2 Implementation

We start trace inlining by computing the maximum trace size that should be inlined at the current call site. This mainly depends on the call site's relevance (see Section 5.1) for program execution. Then, we use a heuristic to decide if it is worth to inline the invoked traces at the current call site. To a large degree, this depends on the size of the traces because inlining large traces causes code bloat.

Inlining method traces is similar to method inlining except that the traces usually do not cover all bytecodes of the callee. So, we build a trace graph from the traces that should be inlined and replace the method invocation with the contents of that trace graph. Then, return instructions that are located within the inlined bytecodes are replaced with direct jumps to the next instruction after the call and exception-throwing instructions are wired to exception handlers located in the caller trace.

**Fig. 4:**
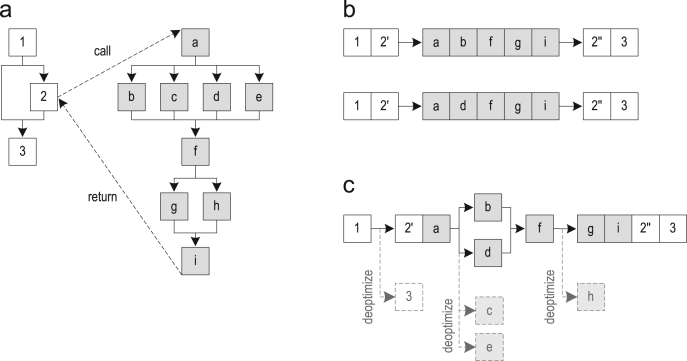
Inlining method traces: (a) control flow graphs; (b) recorded traces; and (c) trace graph after trace inlining.

Fig. 4(a) shows the control flow graphs of two methods. Two traces through those methods are shown in Fig. 4(b). After performing trace recording frequently enough, the recorded traces are getting compiled. The resulting trace graph after trace inlining (but without explicit control flow duplication) is shown in Fig. 4(c). This trace graph is then compiled to optimized machine code. If one of the removed blocks must be executed later on, the compiled code deoptimizes to the interpreter.

Another interesting aspect is that we also remove the edge from block 1 to block 3 although the trace graph does contain block 3. This is advantageous because it avoids control flow merges, which otherwise could constrain compiler optimizations. So, removing edges that are not executed results in better optimized machine code.

In most cases, we inline only those traces that were invoked by the current caller. However, if the callee traces were compiled before trace recording was started for the caller, the caller does not know which of the compiled traces it needs. In those cases, we conservatively consider all callee traces as inlining candidates, except those for which we can prove that they cannot be invoked by the current caller because of the specific parameters that the caller passes to the callee. The used technique behind that is similar to dead code elimination in a method-based compiler but allows eliminating whole traces instead of basic blocks. To further reduce the number of inlined traces, we do also filter out infrequently executed traces (see Section 4.5).

For virtual method invocations, we combine the recorded trace information with the Java HotSpot client compiler's CHA to determine the exact receiver class for the current call site. If the CHA identifies a single target method, the invoked method traces are inlined in a similar way to how the Java HotSpot client compiler inlines methods. If the CHA finds multiple possible target methods, we try to use the recorded receiver classes for inlining the method traces aggressively. For this, we add a run-time check that compares the actual receiver type with the expected type and deoptimizes to the interpreter if the types do not match. By combining CHA and context-sensitive trace information, we can inline virtual calls more frequently than most method-based compilers while emitting run-time checks only where necessary.

**Fig. 5:**
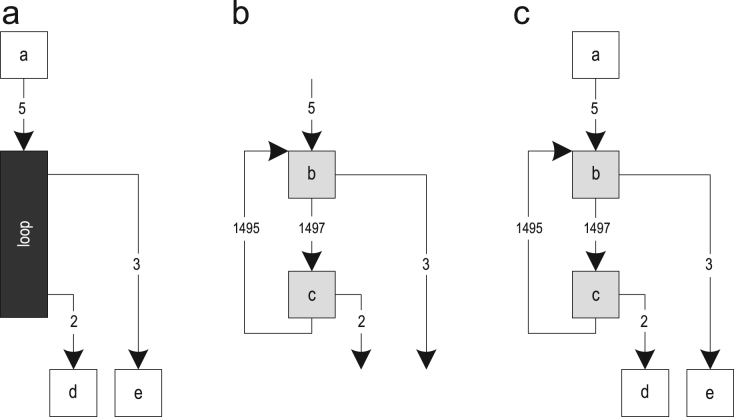
Inlining loop traces: (a) method trace graph; (b) loop trace graph; and (c) after loop inlining.

In addition to inlining method traces, we also support inlining loop traces. Fig. 5(a) shows a trace graph that was built for method traces that invoked loop traces. The loop traces were not inlined yet, so the loop is represented as a black box that is still unknown to the compiler. In the next step, a separate trace graph is built from the loop traces as shown in Fig. 5(b). The actual inlining then replaces the black box in the caller trace graph with the loop trace graph and links all loop exits to their correct successor blocks using jump instructions. In this example, block *b* is linked to block *e* and block *c* is linked to block *d*, resulting in the trace graph shown in Fig. 5(c).

When inlining loop traces, we consider all traces that were recorded for a specific loop as inlining candidates. This is necessary because loop traces are never explicitly linked to their caller trace, so no context-specific call information is available. However, we use the information about the parameters and locals that flow into the loop to eliminate those traces for which we can prove that they cannot be invoked by the current caller. Furthermore, we also eliminate traces that were not executed frequently enough.

A more difficult case is that the inlined loop can have a loop exit for which no successor exists in the caller trace graph. For example, in Fig. 5(a), block *d* could be missing because it was never recorded. However, both loop exits could still be present in the recorded loop traces as shown in Fig. 5(b). One way how this can happen is when the loop traces are compiled before trace recording is started for the method trace. Previously [15], we addressed this issue by explicitly adding deoptimization points for all loop exits that could not be linked to a successor, so that execution deoptimized to the interpreter when such a loop exit was taken. Now, we simply eliminate loop traces that end in a loop exit that is unknown to the current caller. This reduces the number of inlining candidates and results in less generated machine code.

### 4.3 Context sensitivity

Our trace recording infrastructure restricts traces to span at most one method so that the trace-based compiler heavily relies on aggressive trace inlining [15]. The trace recording mechanism preserves context-sensitive information over method boundaries so that each caller knows which parts of the callee it should inline. This helps the compiler to avoid inlining of method parts that were executed frequently in total, but are irrelevant for the current caller. It reduces the generated amount of machine code, and decreases the number of merge points, which increases peak performance.

Also method-based compilers use profiling information to remove never executed code. However, their profiling information typically lacks the context-sensitivity so that they cannot decide which method parts are required for each specific caller. Context-sensitive profiling information could in principle also be recorded for a method-based compiler but we believe that trace recording and trace-based compilation simplify it.

**Fig. 6:**
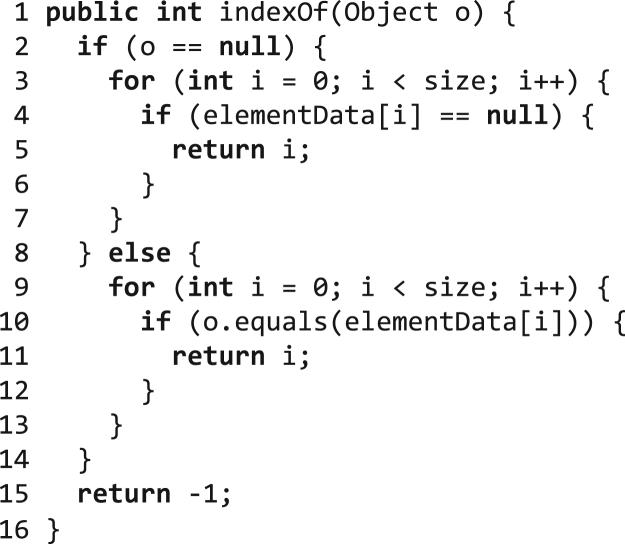
Method ArrayList.indexOf().

Fig. 6 shows the method indexOf() of the JDK class ArrayList. The first part of the method handles the rare case of searching null, while the second part searches the list for non-null objects. Most callers will only require the second part of the method. However, if there is at least one caller in the application that executes the first part of the method, the profiling information in a method-based compiler would indicate that the first part has been executed. So, whenever the method-based compiler inlines the method indexOf(), it does also inline this rarely executed method part. Due to our context-sensitive trace information, our trace-based compiler can avoid that if the caller does not need that specific method part.

Because trace inlining is more selective in what it does inline, our trace-based compiler can use a more aggressive inlining policy, i.e., it can inline traces through methods that would be too large to be inlined as a whole. This increases the compilation scope without necessarily inlining a higher number of Java bytecodes than a method-based compiler. Especially, for complex applications, this results in better optimized machine code and has a significant positive effect on peak performance.

**Fig. 7:**
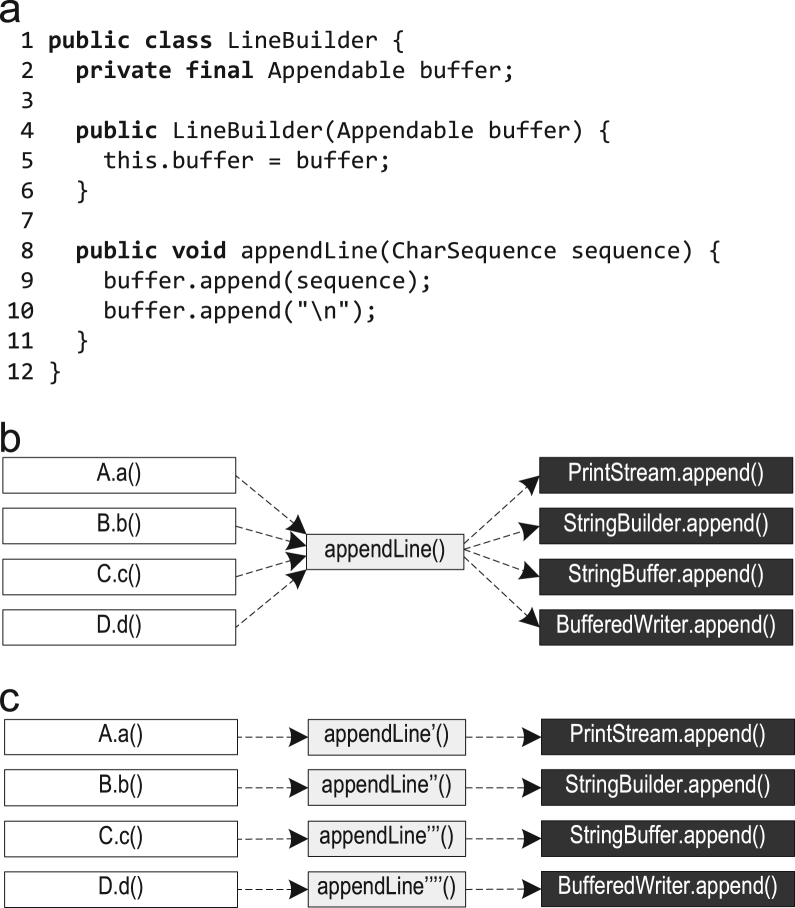
Context-sensitive type information: (a) code pattern; (b) possible method invocations; (c) preferred inlining.

Fig. 7(a) shows the class LineBuilder that wraps an Appendable object and provides the method appendLine(). If multiple LineBuilder objects are used to wrap instances of different classes, such as PrintStream, StringBuilder, StringBuffer, and BufferedWriter, then the invocations of append() on lines 9 and 10 will be polymorphic calls that cannot be inlined easily, as shown in Fig. 7(b).

If the dispatch in appendLine() depends on its call site, e.g., because different LineBuilder objects are used at different call sites, the inlining in Fig. 7(c) would be preferable. Our context-sensitive trace information also stores the receiver types of virtual calls. So, our trace-based compiler can do the preferable inlining indicated in Fig. 7(c) by using this context-sensitive information for aggressive inlining of virtual calls. If a compiler does not record the profiling information in a context-sensitive way, but just accumulates all encountered types (i.e., PrintStream, StringBuilder, StringBuffer, and BufferedWriter at buffer.append()) it will not have enough information to inline such virtual calls.

**Fig. 8:**
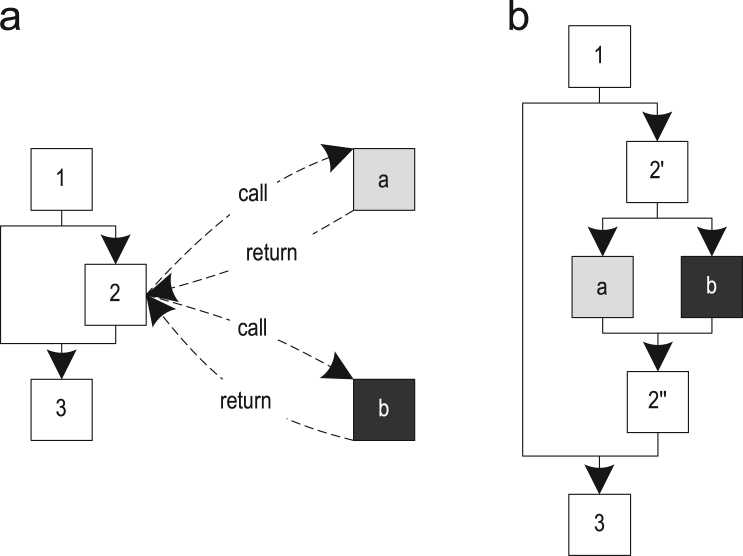
Polymorphic inlining: (a) polymorphic call and (b) polymorphic inlining.

In the previous version of our trace-based compiler, we only used the type information when the recorded traces indicated that the invoked method always belonged to the same type of receiver. Such inlined traces are guarded by a *type guard* that compares the actual receiver type to the expected receiver type and deoptimizes to the interpreter if the types do not match. For this paper, we extended trace inlining in the following ways:

• If a call site always invokes the same method but does it on different receiver types, it was previously not possible to inline the method. However, this occurs frequently, for example, if an abstract base class implements a method that is not overridden by subclasses. We enabled this kind of inlining by guarding it with a so-called *method guard* that accesses the virtual method table of the actual receiver and compares the invoked method with the expected method. If the methods do not match, we deoptimize to the interpreter.
• If a call site always invokes the same method but does it via a receiver of an interface type, we extend type guards to a switch-like structure so that they can check for multiple receiver types. This is cheaper than the interface lookup and allows us to inline invocations of interface methods in many cases. If the actual receiver type does not match any of the expected types we deoptimize to the interpreter.
• Another enhancement is the inlining of polymorphic calls. Fig. 8(a) shows a method, where a virtual call might invoke two different methods. Because these methods are small, it pays off to inline them both. This results in the control flow shown in Fig. 8(b) where block 2′ dispatches to one of the inlined methods depending on the type of the actual receiver. Here, we also use switch-like semantics so that several types can dispatch to the same inlined method. If the actual receiver type does not match any of the expected types, we deoptimize to the interpreter.

The Java HotSpot server compiler also inlines polymorphic calls but limits the number of inlined methods to at most two, as a higher number could easily result in code bloat. Our trace-based compiler inlines method parts more selectively due to the context-sensitivity of the recorded traces. So, we can avoid inlining of method parts that were executed frequently in total, but are not required for the current caller. Furthermore, the recorded type information is also context-sensitive, which reduces the number of inlining candidates. So, our trace-based compiler does not have to limit the number of inlined methods but instead only limits the total size of all inlined methods depending on the execution frequency of the specific call site. For applications with a high number of polymorphic calls, this results in significantly better inlining and therefore a higher performance, while avoiding issues with code bloat.

### 4.4 Native methods

Java code can call native methods using the Java Native Interface (JNI). This mechanism is mainly used to implement platform-specific features that could not be expressed in Java otherwise. Some methods of the Java standard library, e.g., System.arraycopy(), are implemented in a platform-specific way directly in the JVM. As no Java code is executed for such methods, trace recording is not possible for those methods.

The Java HotSpot VM uses compiler intrinsics for the most important platform-specific methods so that the JIT compiler can inline such methods. If our trace-based JIT compiler compiles a trace graph that contains the invocation of a native method that is implemented as a compiler intrinsic, we do exactly the same inlining as the method-based compiler. Still, our trace-based compiler has one advantage: traces are smaller than methods so that our trace-based compiler can inline Java traces more aggressively than a method-based compiler could inline Java methods. This results in a larger compilation scope so that the caller of a native method has specific knowledge about the parameters that are passed to the native method. The JIT compiler can use this information to optimize inlined compiler intrinsics more aggressively.

**Fig. 9:**
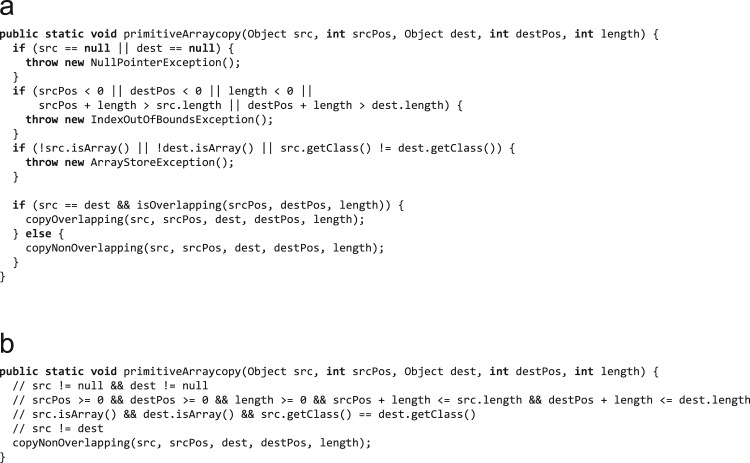
Pseudo-code for System.arraycopy() when copying primitive type arrays: (a) unoptimized and (b) optimized.

Fig. 9(a) shows pseudo-code for the implementation of the native method System.arraycopy(), which is used to copy primitive type arrays. Depending on the compiler's information about the parameters that are passed to System.arraycopy(), it can optimize the intrinsic. Fig. 9(b) shows an optimized version of the method where the compiler could optimally exploit the parameter values. The necessary parameter information is for example available when the source and the destination arrays are allocated in the same compilation scope in which System.arraycopy() is inlined. So, increasing the compilation scope can help to increase the performance of inlined compiler intrinsics.

### 4.5 Filtering out traces

**Fig. 10:**
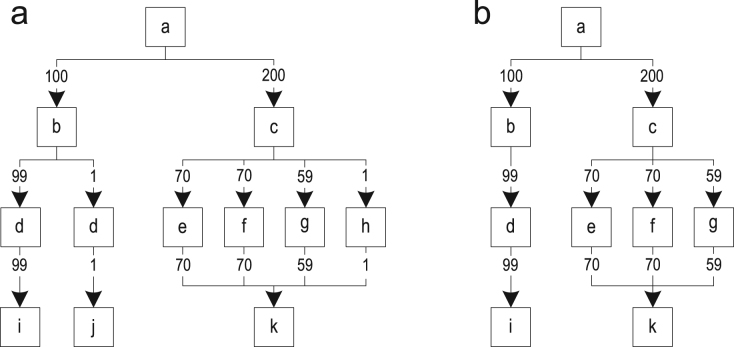
Filtering out infrequently executed traces: (a) original trace graph and (b) trace graph after filtering.

When a trace is recorded, chances are good that the trace is important and will be executed frequently. Still, sometimes recorded traces turn out to be rarely executed. By eliminating such traces, we can ensure that only important paths are compiled. Fig. 10(a) shows the trace graph after merging all recorded traces. The graph edges are annotated with the execution frequencies.

For every block, we determine the most frequently executed outgoing edge and compare its frequency to those of all other outgoing edges of the same block. Then, we remove all edges with a 100× lower execution frequency. After processing all blocks, we remove no longer reachable blocks from the trace graph. Fig. 10(b) shows the resulting graph after filtering.

The recorded trace information conserves the program behavior that was observed during a specific time frame. At a later point of execution, infrequently executed (and therefore eliminated) paths might become important as the program behavior may change over time. This results in frequent deoptimization because not compiled paths get to be executed. If too frequent deoptimization is detected, the compiled machine code is invalidated and another compilation is triggered. This compilation avoids trace filtering for those cases that resulted in frequent deoptimization.

Trace filtering has the following corner cases, where extra care must be taken:

• For most loops, the loop body is executed significantly more frequently than the loop exits, see Fig. 5(c). So, the execution frequencies of the loop exits have to be compared to the frequency of the loop entry instead of to the frequency of the backward branch. Otherwise, the loop exit edges would be filtered out, so that deoptimization to the interpreter is required after executing a loop. This would increase the deoptimization frequency and it would limit the possible compilation scope.
• Aggressive trace inlining may also inline infrequently executed traces. Those inlined traces may not necessarily reflect the typical execution behavior yet so that trace filtering might eliminate important traces. This would result in frequent deoptimization so that trace filtering should be avoided for insufficiently trace-recorded methods and loops.

## 5 Trace inlining heuristics

All inlining heuristics that are presented in the following section have in common that they first compute the relevance of a call site and then use that relevance to compute the maximum inlining size. The actual inlining decision is a simple comparison of the maximum inlining size with the actual size of the traces that should be inlined. For our evaluation, we paired several inlining heuristics with different relevance computation algorithms.

### 5.1 Relevance of a call site

**Fig. 11:**
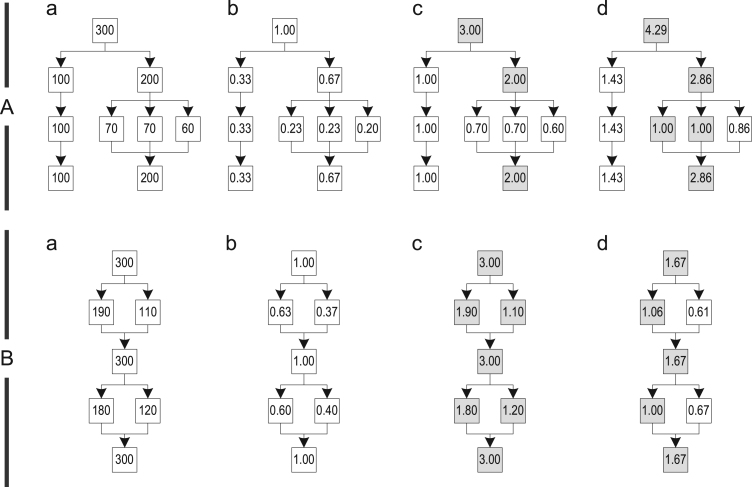
Different relevance computation algorithms: (a) node execution counts; (b) simple; (c) most frequent trace; and (d) path-based.

The relevance of a call site is determined by the relevance of the trace graph block in which the call site is located. We evaluated three different algorithms for computing the relevance and illustrate their behavior on the two trace graph examples A and B shown in Fig. 11. Example A was built from four different traces that hardly share any blocks. Example B also shows a trace graph built from four traces, but every block is shared with at least one other trace.

For computing the relevance of the trace graph blocks, we first determine how often each block was executed by recorded traces. Fig. 11(a) shows the trace graphs where every block is annotated with its execution frequency. Then, we compute the relevance of each block by dividing its execution frequency with a reference value. Depending on the reference value, the relevance is scaled differently. So, we use one of the following algorithms to choose that reference value:

• *Simple*: The simplest way of computing the relevance of a trace graph block is to divide its execution frequency by the total execution frequency of all traces merged into the trace graph. The resulting value is in the range ]0, 1] and assigns a high relevance to those blocks in which inlining has a positive effect during most executions, as shown in Fig. 11(b).
• *Most frequent trace*: Another way is to divide the block execution frequency by the execution frequency of the most frequently executed trace ever merged into the trace graph. Because traces are merged, trace graph blocks that are shared between multiple traces have a higher execution count than they would have without merging. So, this metric returns a high relevance for call sites that are within such shared blocks, while returning a value in the range ]0, 1] for call sites that are only contained in individual traces. In Fig. 11(c), the colored blocks are shared and therefore get a higher relevance. If many different traces were recorded and many blocks are shared in the trace graph, then it can happen that every block in the trace graph has a relevance greater than 1, as shown in example B of Fig. 11(c).
• *Path-based*: Our third approach computes a variant of the most frequently executed path through the trace graph. We start at the root block of the trace graph and determine the most frequently executed successor block. Then, we mark this block as visited and continue with this block recursively until we either reach a block without successors or we are back at the loop header. All blocks that are visited due to this algorithm are colored in Fig. 11(d). Then, we use the lowest execution frequency of all visited blocks to compute the relevance of all other blocks in the trace graph. This has the advantage that important call sites, i.e., those on this path and on frequently executed split/merge points, have a value in the range [1,∞[, while less important calls have a value in the range ]0, 1[.

### 5.2 Configurations

We started with 15 different inlining heuristics ranging from static heuristics to dynamic ones. For each inlining heuristic, we performed a systematic search to find good parameter settings. During our evaluation, our dynamic inlining heuristics outperformed all static ones so that we omit detailed results for static inlining heuristics in this paper. Furthermore, we describe only those variants of our dynamic inlining heuristics that showed a good peak performance or a small amount of generated machine code.

• *Minimum code*: This heuristic modifies an inlining size of 35 bytecodes based on the relevance of the call site. A relevance below 1 reduces the inlining size, while a relevance greater than 1 increases the inlining size. By combining this heuristic with the *path-based* relevance computation algorithm, it shows a fairly good peak performance while generating small amounts of machine code. We also tried combining this inlining heuristic with the relevance computation algorithm *simple*. However, this has a significant negative effect on the peak performance while generating only slightly less machine code. Therefore, we omit detailed results for this second variant.
• *Balanced*: This heuristic increases an inlining size of 40 bytecodes based on the relevance of the call site. A relevance below 1 does not affect the inlining size, while a relevance greater than 1 increases the inlining size. So, decreasing the predefined size is explicitly not allowed, which makes it more likely that important calls are inlined. We use this heuristic with the *path-based* relevance computation algorithm, which results in a balance between peak performance and amount of generated machine code.
• *Performance*: This inlining heuristic uses a large inlining size of 150 bytecodes and decreases that for call sites with a relevance below 1. Increasing the inlining size beyond the predefined value is explicitly not allowed. For computing the relevance of the call sites, we again use the *path-based* relevance computation algorithm. So, this heuristic is optimized for peak performance while generating still reasonably small amounts of machine code.
• *Greedy*: Similar to the previous configuration, this heuristic uses a very large inlining size of 250 bytecodes and decreases it for call sites with a relevance below 1. To ensure that called traces are inlined greedily, we combine this heuristic with the *most frequent trace* relevance computation algorithm. However, due to the predefined maximum value, even this inlining heuristic avoids inlining of huge traces. This heuristic shows which of the benchmarks described in Section 6 profit from very aggressive trace inlining. We also experimented with even more aggressive inlining heuristics but those did not further improve the peak performance, while generating more machine code.

Similar to method-based compilers, all our heuristics make sure that tiny methods such as accessors are always inlined. This makes sense, because invoking small traces may require more machine code than the inlining. Another strategy, that is used by the Java HotSpot server compiler, is to avoid method inlining if the callee was already compiled separately and the compilation resulted in a large amount of generated machine code. This assumes that a fairly large compilation scope has already enough information for good compiler optimizations so that increasing the compilation scope beyond a certain point is not useful. We also use this technique for all our trace inlining heuristics as it reduces the generated machine code without affecting the performance measurably.

To reduce the probability of nested trace inlining, we ensure that inlined traces inherit the relevance from their parent call site. For this, we multiply the relevance of every callee block with the relevance of the caller's block. However, we limit the maximum inherited relevance to 1 as the relevance could otherwise increase with the inlining level. Relevance inheriting again reduces the amount of generated machine code without affecting the performance measurably and is also used by all our heuristics.

## 6 Evaluation

We implemented our trace-based JIT compiler for the IA-32 architecture of Oracle's Java HotSpot VM using the early access version b12 of the upcoming JDK 8 [20]. For evaluating our inlining heuristics, we chose the benchmark suites SPECjbb2005 [23], SPECjvm2008 [24], and DaCapo 9.12 Bach [3] as those cover a large variety of benchmarks. The benchmarking system has the following configuration: an Intel Core-i5 processor with 4 cores running at 2.66 GHz, 4⁎256 kb L2 cache, 8 MB shared L3 cache, 8 GB main memory, and with Windows 7 Professional as the operating system.

The results are shown relative to the results for the unmodified, method-based Java HotSpot client compiler, which is denoted by 100%. For the trace-based JIT compiler, the amount of generated machine code also includes data that is specific to trace-based compilation such as additional deoptimization information required for fall back to the interpreter. Each benchmark suite was executed 10 times and we report the average of those results along with the 95% confidence interval.

### 6.1 SPECjbb2005

The SPECjbb2005 benchmark simulates a client/server business application where all operations are performed on an in-memory database that is partitioned into so-called warehouses. The benchmark is executed with different numbers of warehouses, and each warehouse is processed by one thread. We use a system with 4 cores for benchmarking so that the official SPECjbb2005 throughput in business operations per second (bops) is defined as the geometric mean of the performance for the warehouses 4–8. A heap size of 1200 MB is used for all measurements.

**Fig. 12:**
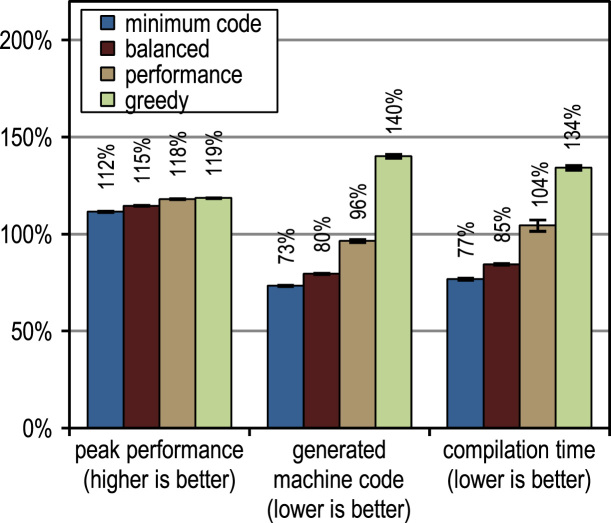
SPECjbb2005 results.

Fig. 12 shows the peak performance, the generated machine code and the compilation time for the SPECjbb2005 benchmark. All our trace-based compiler variants outperform the client compiler significantly in terms of peak performance. More aggressive trace inlining results in a higher performance but does also generate more machine code and requires a longer compilation time because of the larger size of the compilation units. The peak performance of the SPECjbb2005 benchmark clearly profits up to the configuration *performance* from the increased trace inlining aggressiveness. Our configuration *greedy* increases the performance only slightly, while generating significantly more machine code. In terms of compilation time and amount of generated machine code, our configuration *minimum code* is especially efficient, while reaching a decent peak performance.

**Fig. 13:**
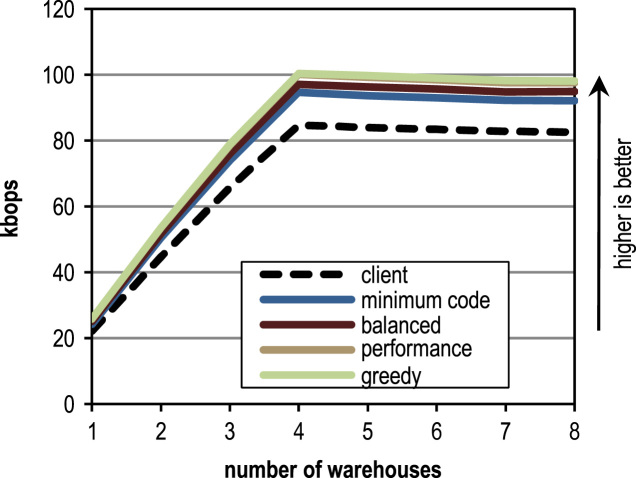
SPECjbb2005 peak performance for different numbers of warehouses.

Fig. 13 shows the SPECjbb2005 peak performance for different numbers of warehouses and different inlining heuristics. The maximum peak performance is reached with 4 warehouses as every warehouse is processed by one thread and our benchmarking system has 4 cores. The figure shows that our tracing configurations outperform the method-based client compiler independently of the used number of warehouses.

### 6.2 SPECjvm2008

The SPECjvm2008 benchmark consists of 9 benchmark categories that measure peak performance. Next to the individual benchmark results, we present the geometric mean of all results. A heap size of 1024 MB is used for all measurements.

**Fig. 14:**
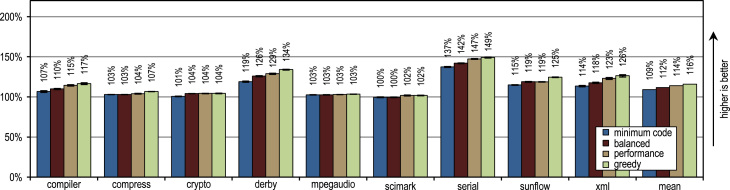
SPECjvm2008 peak performance.

**Fig. 15:**
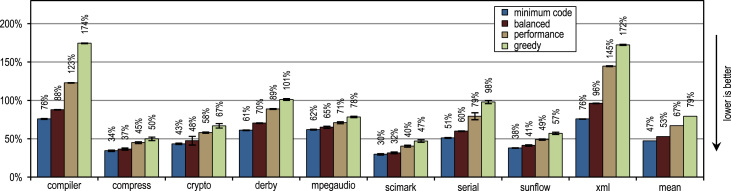
SPECjvm2008 generated machine code.

**Fig. 16:**
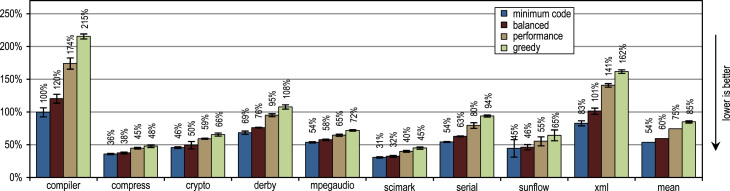
SPECjvm2008 compilation time.

Fig. 14 shows that all our tracing configurations outperform the method-based HotSpot client compiler. Our tracing configurations show the highest speedups on the benchmarks *derby* and *serial*. There, trace inlining achieves a larger compilation scope than the method inlining used by the HotSpot client compiler. A very aggressive trace inlining policy such as our configuration *greedy* does increase the peak performance especially for the benchmarks *derby* and *sunflow*. However this aggressive trace inlining also increases the amount of generated machine code and the time required for JIT compilation as shown in Fig. 15, Fig. 16.

The small and loop-intensive benchmarks *crypto*, *mpegaudio*, and *scimark* show almost no increased peak performance because the method-based HotSpot client compiler can inline all calls in the performance critical loops as well. Due to the small size of these benchmarks, our trace-based compiler can achieve only a similar-sized compilation scope as the method-based compiler. However, our trace-based compiler inlines traces instead of whole methods so that significantly less machine code is generated and less compilation time is required, as shown in Fig. 15, Fig. 16.

Fig. 15 shows that the amount of generated machine code is sensitive to the inlining size, so that it can easily happen that lots of machine code is generated. However, small and loop-intensive benchmarks do not show a large increase in code size, even when our most aggressive trace inlining heuristic is used. On such benchmarks, a trace inlining heuristic can only be too conservative but never too aggressive.

### 6.3 DaCapo

The DaCapo 9.12 Bach benchmark suite consists of 14 Java applications. Using the default data size and a heap size of 1024 MB, each benchmark is executed for 20 iterations so that the execution time converges. We present the fastest run for each benchmark and the geometric mean of all results. A heap size of 1024 MB is used for all measurements.

**Fig. 17:**
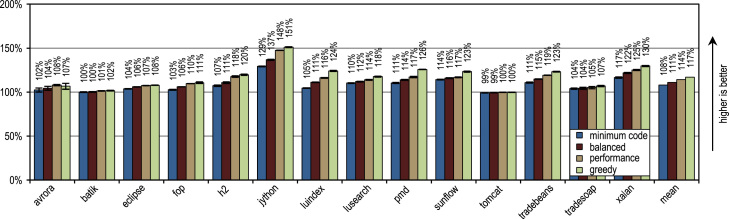
DaCapo 9.12 Bach peak performance.

**Fig. 18:**
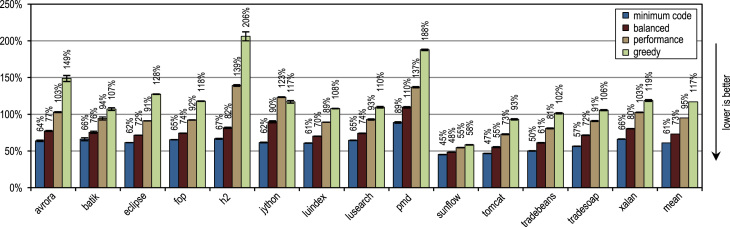
DaCapo 9.12 Bach generated machine code.

Fig. 17 shows the peak performance results for the DaCapo 9.12 Bach benchmarks, while Fig. 18 shows the amount of generated machine code. The inlining heuristic *greedy* shows the best peak performance, while generating more machine code than the unmodified Java HotSpot client compiler. Especially the benchmarks *luindex*, *pmd*, and *sunflow* profit from the *greedy* heuristic because of its large inlining size. However, on some of the benchmarks this heuristic is already over-aggressive so that more machine code is generated without a measurable change in peak performance.

The tracing configurations achieve the highest speedup for the benchmark *jython*, which executes a large number of virtual calls. Our trace-based compiler uses the recorded trace information for aggressive inlining of virtual calls. So, it achieves a large compilation scope, which results in a high peak performance.

On average, all inlining heuristics except *greedy* generate less machine code than the method-based client compiler, while still showing a higher average peak performance. One of our static inlining heuristics, which only compares the size of the traces to a fixed maximum value, reached nearly the same peak performance as *minimum code* but generated more machine code. This good result for a static inlining heuristic comes from the fact that the compiled traces contain only frequently executed paths per definition. So, for methods without much control flow, it is sufficient to compare the size of the traces to a fixed threshold. As long as the maximum inlining size was kept low, this static heuristic showed decent results but when the maximum inlining size was increased, lots of machine code was generated without a significant positive effect on peak performance.

**Fig. 19:**
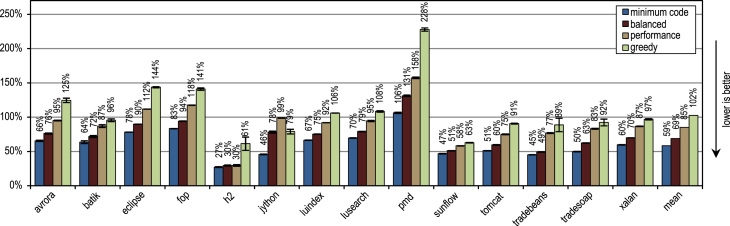
DaCapo 9.12 Bach compilation time.

In terms of time required for JIT compilation, Fig. 19 shows that our trace-based compiler is highly efficient so that even our most aggressive configuration *greedy* requires on average only a similar amount of time as the method-based HotSpot client compiler.

### 6.4 High-level optimizations

**Fig. 20:**
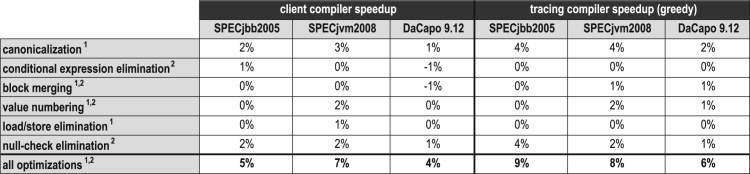
Impact of high-level optimizations on peak performance.

Similar to method-inlining, trace inlining is an optimization that has positive effects on other compiler optimizations due to the larger compilation scope. Fig. 20 compares the impact on peak performance for different high-level optimizations that are used by both the method-based HotSpot client compiler and our trace-based compiler. Optimizations marked with ^1^ are local optimizations that are applied directly during bytecode parsing, while optimizations marked with ^2^ are global optimizations that run in a separate phase after graph building.

In general, aggressive trace inlining increases the compilation scope, which positively affects many optimizations. However, depending on the benchmarks the individual optimizations show different gains. For the SPECjbb2005 benchmark, our trace inlining especially increases the effectiveness of *canonicalization*, which performs simple optimizations such as constant folding and dead code elimination. The SPECjvm2008 benchmark suite contains too many small and loop-intensive benchmarks where our trace-based compiler cannot achieve a significantly larger compilation scope. Therefore, hardly any high-level optimization profits significantly here. On the DaCapo 9.12 Bach benchmark suite, the performance increase is distributed over all listed optimizations.

### 6.5 Server compiler

Oracle's Java HotSpot VM has two different JIT compilers that share most parts of the VM infrastructure. Our trace-based compiler builds on the *client compiler*, which is designed for best startup performance and implements only basic optimizations to achieve a decent peak performance. The *server compiler* is designed for long-running server applications and performs significantly more optimizations to produce highly efficient code. Some additional optimizations that the server compiler performs are array bounds check elimination, loop-invariant code motion, loop unrolling, and escape analysis. Thus, the server compiler generates code with a better peak performance but requires a 6–31 times (13 times on average) longer compilation on the benchmark suites SPECjbb2005, SPECjvm2008, and DaCapo 9.12 Bach. A server executes mostly long-running applications so that the long compilation time is only a small overhead in comparison to the total execution time.

**Fig. 21:**
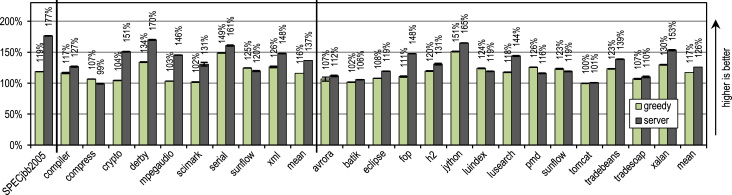
SPECjbb2005, SPECjvm2008, and DaCapo 9.12 Bach peak performance.

We compared our best peak performance configuration *greedy* to the HotSpot server compiler, and the results are shown in Fig. 21. The SPECjbb2005 benchmark profits heavily from some of the time-consuming optimizations of the server compiler, so that our trace-based compiler reaches only 67% of the server compiler's performance.

For the SPECjvm2008 benchmark suite, our tracing configuration *greedy* reaches on average 85% of the server compiler's performance. This benchmark suite contains many loop-intensive benchmarks, such as *crypto*, *mpegaudio*, and *scimark*, where the server compiler shows a significantly higher peak performance, because it applies array bounds check elimination and sophisticated loop optimizations. However, due to our aggressive trace inlining our trace-based compiler outperforms the server compiler on the benchmarks *compress* and *sunflow*.

On the DaCapo 9.12 Bach benchmark suite, which contains significantly more complex and less loop-intensive benchmarks [14], our trace-based compiler reaches 93% of the server compiler's peak performance on average. However, although our trace-based compiler performs only basic compiler optimizations, it outperforms the server compiler's peak performance on the benchmarks *luindex*, *pmd*, and *sunflow*. This is due to our aggressive and context-sensitive trace inlining approach.

### 6.6 Startup performance

The Java HotSpot client and server compilers as well as our trace-based JIT compiler are all designed for multi-threaded background compilation. So, we evaluate the startup performance in the following scenarios:

• The first scenario executes 1 *application thread*, while the VM uses up to 4 JIT compiler threads. So, on our 4 core benchmarking system, up to 3 cores can be exclusively used for JIT compilation.
• In the second scenario, 4 *application threads* are executed while the VM uses up to 4 JIT compiler threads. On our 4 core benchmarking system, the JIT compiler threads compete with the application threads. The Java HotSpot VM assigns a higher priority to JIT compiler threads as early JIT compilation has a positive effect on startup performance.

All presented results are normalized to the performance of the configuration *client* with 1 JIT compiler thread. We do not present any startup performance results for the SPECjbb2005 benchmark as this benchmark is designed to measure peak performance so that first results are obtained after 30 s where all configurations are already close to their peak-performance.

**Fig. 22:**
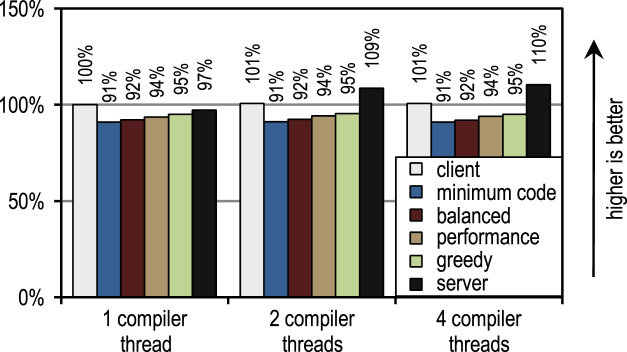
SPECjvm2008 startup performance with 1 application thread.

**Fig. 23:**
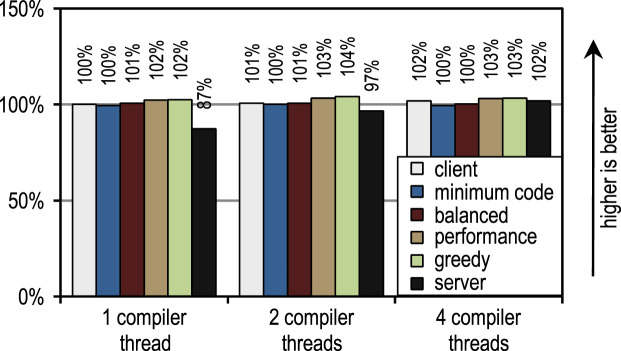
SPECjvm2008 startup performance with 4 application threads.

For the SPECjvm2008 benchmark suite, we measured the startup performance by executing one operation for each benchmark. Fig. 23 shows that the HotSpot client compiler and our trace-based compiler achieve good results for those scenarios where the application threads compete with compiler threads so that compilation time matters. Fig. 22 shows the different scenario when JIT compilation can be offloaded to otherwise idle cores. There, the HotSpot server compiler shows the best startup performance because the SPECjvm2008 benchmark suite contains several small benchmarks where there is little to compile and which almost reach their peak performance after compiling the innermost benchmark loop. This is the ideal case for the server compiler which does optimize loops especially well so that its peak performance advantage also affects the startup performance results if idle cores are available for compilation.

The figures also show that the number of compiler threads does neither affect the client compiler nor our trace-based compiler because both JIT compilers require little time for compilation. The server compiler requires much time for compilation and therefore greatly profits from more than one compilation thread, especially if there are idle cores that can be used.

**Fig. 24:**
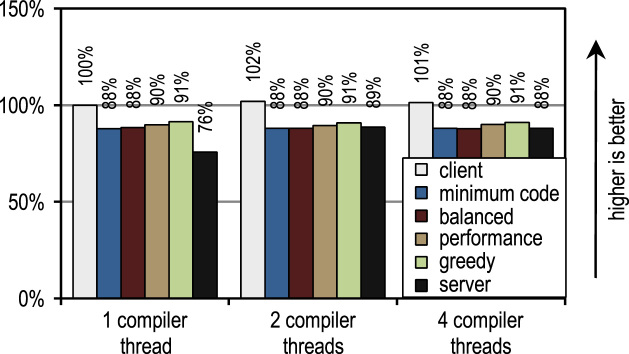
DaCapo 9.12 Bach startup performance with 1 application thread.

**Fig. 25:**
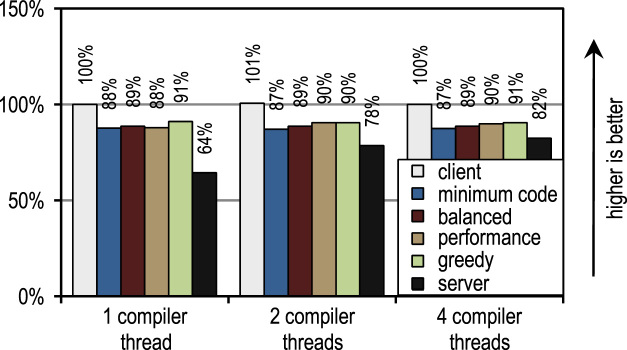
DaCapo 9.12 Bach startup performance with 4 application threads.

For the DaCapo 9.12 Bach benchmark suite, we measured the startup performance by executing one iteration for each benchmark. The benchmark results are shown in Fig. 24, Fig. 25.

Again, the number of compiler threads neither affects the client compiler nor our trace-based compiler because those require too little time for compilation. In contrast to that, the server compiler profits when more than one thread is used for JIT compilation. However, several of our tracing configurations always show a higher startup performance than the server compiler, even if the server compiler can use idle cores for JIT compilation. This is the case because the DaCapo 9.12 Bach benchmarks are significantly more complex than the SPECjvm2008 benchmarks [14], so that the JIT compilation performance is the dominating factor for startup performance.

So, on the DaCapo 9.12 Bach benchmarks, our trace-based configurations show a roughly 10% slower startup than the HotSpot client compiler. While our trace-based compiler often requires even less time for JIT compilation than the HotSpot client compiler, it shows a lower startup performance because of the additional overhead for trace recording and deoptimization that mainly incur during startup. However, this drawback is outweighted by the significantly improved peak performance. Unlike the HotSpot server compiler, our trace-based compiler does not need to use idle cores to achieve a good startup performance.

## 7 Related work

Bala et al. [1] implemented trace compilation for their Dynamo system to optimize native instruction streams. Hot instruction sequences are identified by executing the binary application in an interpreter. Then, those traces are compiled and the generated machine code is executed directly. The interpretation overhead decreases with the number of compiled traces, resulting in a speedup eventually. In contrast to them, we identify suitable trace anchors within Java bytecodes for which we use static analysis during class loading. We limit individual traces to at most one method and instead link the recorded traces to preserve context-sensitive trace information over method boundaries. This allows us to delay the inlining decision to the time of compilation instead of doing it already during trace recording.

Rogers [22] implemented a JIT compiler for Java where frequently executed basic blocks are detected and compiled. Related blocks, which may also span multiple methods, are grouped and optimized as an entity when executed frequently. Compared to method-based compilation, up to 18% fewer bytecodes are compiled. Our system records and compiles traces and uses context-sensitive trace inlining to increase the peak performance while at the same time generating only modest amounts of machine code.

The next approaches implemented different variants of trace-based compilation for Java [2], [11], [12], [18]. However, all approaches have in common that traces may span more than one method, so that inlining must be performed during trace recording. In contrast to that, we assume that one method is the maximum scope of a trace and use trace linking to preserve call information between traces. This allows delaying the inlining decision to the time of compilation when more information is available. So, our inlining can be more selective while using simple inlining heuristics that result in a peak performance increase and a reduced amount of generated machine code.

Gal et al. [11], [12] implemented trace-based compilation for Java on resource-constrained devices. Traces start at frequently executed backward branch targets and side exits of existing traces. Each trace may span multiple methods so that inlining is performed during trace recording. After recording a trace, it is compiled to machine code and linked to other compiled traces to form a tree-like structure. The simple structure of the compiled traces simplifies many optimizations but may result in excessive tail duplication and code bloat. A similar approach is also used by the Dalvik VM [4], [6] on Android-based mobile devices. In contrast to that, we merge individual traces into a trace graph before compilation to avoid excessive tail duplication.

Bebenita et al. [2] implemented trace-based compilation for the Maxine VM. The Maxine VM uses a baseline JIT compiler instead of an interpreter for the initial executions. This baseline JIT compiler was modified to generate the trace recording instrumentation. Prior to JIT compilation, the recorded traces are merged into trace regions which have explicit control flow merge points. This avoids unnecessary tail duplication. Due to various loop optimizations, the JIT compiler achieves excellent speedups for loop-intensive benchmarks. However, it performs worse than method-based compilation on benchmarks with fewer loops. Our work is complementary as it focuses on complex applications that are not loop-intensive such as DaCapo 9.12 Bach *jython*. We achieve excellent speedups for those applications, while loop-intensive benchmarks only show small speedups as our trace-based compiler does not perform any sophisticated loop optimizations yet.

Inoue et al. [18] added a trace-based JIT compiler to the IBM J9/TR JVM by modifying the method-based JIT compiler. They record linear and cyclic traces without any inner join points and compile them to optimized machine code. In terms of peak performance, their trace-based compiler nearly reaches the performance of the similarly optimizing method-based JIT compiler. Wu et al. [28] extended that work by avoiding short-lived traces and unnecessary trace duplication. While this does not affect peak performance, it reduces both the amount of generated machine code and the compilation time. We also build on an existing production quality JVM but limit individual traces to span at most one method. So, we can delay the inlining decision to the time of compilation, which increases the peak performance.

Bradel and Abdelrahman [5] used traces for method inlining in the Jikes RVM. The traces are recorded using an offline feedback-directed system. Then, frequently executed call sites are identified within the recorded traces and this information is used to perform method inlining. Their evaluation with the benchmarks SPECjvm98 and Java Grande shows a 10% performance increase, while 47% more machine code is generated. Our system records traces during execution in the interpreter and only compiles and inlines method parts covered by traces. This increases the peak performance and can reduce the amount of generated machine code.

Hazelwood and Grove [16] implemented context-sensitive inlining for a method-based compiler. Timer-based sampling and recording of call information is used to guide inlining decisions during compilation. The amount of generated machine code and the compilation time is reduced by 10% without affecting the performance. In our system, the recorded traces contain even more detailed context-sensitive information. Depending on the inlining heuristic, this increases peak performance or reduces the amount of generated machine code.

Method inlining is a well-researched topic that is extensively covered in the literature. The remaining related work therefore concentrates on ways to inline *method parts* instead of whole methods as this is closest to our work. Still, these approaches are complementary to trace compilation as method parts are explicitly *excluded* there, while trace recording identifies method parts that should be compiled.

Partial method compilation [9], [26] uses profiling information to detect rarely executed method parts in order to exclude them from compilation. This reduces the compilation time, increases the startup performance, and may also have a positive impact on peak performance. Our approach is even more selective as we record and compile only frequently executed traces. Furthermore, we use the saved compilation resources for more aggressive and context-sensitive inlining, which increases the peak performance.

Suganuma et al. [25] implemented region-based compilation where rarely executed method parts are excluded from compilation by using heuristics and profiling information. Then, frequently executed code is grouped into one compilation unit by heavy use of method inlining. This reduces the compilation time by more than 20% and increases the peak performance by 5% on average for the benchmark suites SPECjvm98 and SPECjbb2000. We only compile frequently executed method parts covered by recorded traces and use trace inlining to increase the performance.

## 8 Conclusions

In this paper, we presented a trace-based compiler for Java that performs trace inlining during JIT compilation instead of during trace recording. Traces have the advantage that they cover only the executed method parts. Delaying the inlining decision to the time of JIT compilation allows more selective trace inlining as more information is available at that time. Furthermore, our recorded traces are context-sensitive so that we can inline different method parts depending on the specific call site. This allows very aggressive trace inlining while generating reasonable amounts of machine code. Additionally, we propose to eliminate infrequently executed traces before compilation to ensure that only the most frequently executed traces are compiled to machine code.

The evaluation with the benchmark suites SPECjbb2005, SPECjvm2008, and DaCapo 9.12 Bach showed that good trace inlining can increase the peak performance while generating less machine code than method inlining. Furthermore, we also showed that trace inlining achieves larger compilation scopes that increase the effectiveness of common compiler optimizations and eventually result in a better peak performance.
